# Differences in electron beam dosimetry using two commercial ionization chambers and the TG‐21 protocol: Another reason to switch to TG‐51

**DOI:** 10.1120/jacmp.v4i2.2527

**Published:** 2003-03-01

**Authors:** David S. Followill, William F. Hanson, Geoffrey S. Ibbott, Leon R. Eglezopoulos, Chen‐Shou Chui

**Affiliations:** ^1^ Department of Radiation Physics The University of Texas M. D. Anderson Cancer Center Houston Texas 77030; ^2^ IBA 3150 Stage Post Drive, Suite 110 Bartlett Tennessee 38133; ^3^ Department of Medical Physics Memorial Sloan‐Kettering Cancer Center 1275 York Avenue New York New York 10021

**Keywords:** dosimetry, electron dosimetry, photon dosimetry, parallel plate, megavoltage calibration

## Abstract

Two of the most popular dosimetry systems used for calibration of megavoltage photon and electron beams in radiation therapy are (i) cylindrical Farmer‐type chambers in liquid water and (ii) Holt Memorial parallel‐plate chambers in clear polystyrene. Since implementation of the AAPM TG‐21 calibration protocol, the Radiological Physics Center (which uses the Farmer in‐water system) has compared machine calibrations on two occasions with those of Memorial Sloan‐Kettering Cancer Center (which uses the Holt in‐polystyrene system). Two years post publication of the TG‐51 protocol, 70% of the clinics monitored by the RPC still use TG‐21. Seventeen photon beams from cobalt‐60 to 18 MV and 31 electron beams from 6 to 20 MeV were compared using the TG‐21 protocol. These data represent the most comprehensive comparison of the two most popular systems in use. Based on the average percent difference, the two systems yielded the same absorbed dose to water at the reference point in phantom to within 1.5% for both modalities. No energy dependence was evident in the results; however, a systematic average percent difference between photons and electrons was seen, with the Farmer in‐water system consistently predicting a dose 1.3% lower for electrons than the Holt in‐polystyrene system. For photons both systems predicted the same dose to within 0.3% on average. When a physicist converts from TG‐21 to TG‐51, these data may be of assistance in explaining unexpected changes in output that are different from previously published values. Implementation of the TG‐51 protocol should eliminate any of the observed differences in electron beam dosimetry between the two dosimetry systems because the Holt system cannot be used with TG‐51.

PACS number(s): 87.53.‐j, 87.53.‐j

## I. INTRODUCTION

The Radiological Physics Center (RPC) has relied on Farmer‐type cylindrical ionization chambers in water as its “standard” dosimetry system for more than 25 years.^1^ Physicists at Memorial Sloan‐Kettering Cancer Center (MSK) developed and have used the Holt Memorial Parallel Plate Chamber (MPPK) in a clear polystyrene phantom as their “standard” over the same time period.^2^

In 1980, when the RPC performed a dosimetry review visit to MSK, significant differences (up to 5%) between the two dosimetry systems were observed, particularly for low‐energy electrons. At that time the RPC used the Subcommittee on Radiation Dosimetry (SCRAD)^3^ and International Commission on Radiation Units (ICRU‐21)^4^ calibration protocols for photons and electrons, respectively, while MSK was using a calculation protocol based on the Bragg Gray Cavity Theory, much of which was incorporated into the American Association of Physicists in Medicine (AAPM) protocol TG‐21.^5^ Since 1984, when the RPC and MSK both converted to dosimetry calculations based on the TG‐21 protocol, the RPC has performed on‐site dosimetry review visits at MSK on two occasions. During these reviews, machine calibration and determination of absorbed dose to water under reference conditions were compared for a wide range of photon and electron energies. The results of these measurements are presented here. Four RPC physicists were involved in the measurements, and numerous physicists were responsible for the clinical calibrations at MSK. Four different RPC Farmer‐type ion chamber/electrometer systems and at least five MSK parallel plate chamber/electrometer systems were employed. The results represent, therefore, a robust comparison of the two dosimetry systems under field conditions using the TG‐21 protocol.

Several papers have been published in which the calibration of parallel plate and cylindrical chambers were compared under various conditions,^6^ ^–^ ^10^ generally in the same phantom material. Comparisons of parallel‐plate and cylindrical‐chamber ionization measurements have also been used to investigate various chamber‐specific dosimetry parameters such as $N_{\text{gas}},\;P_{\text{repl}}$, and $P_{\text{pol}}$,^11^ ^–^ ^13^ again often in the same phantom material. However, no detailed comparison of the measured absolute dose in water and polystyrene media for the two most popular dosimetry systems in use in the USA has been reported.

Arguments can be made for and against both dosimetry systems under a variety of circumstances. These include the uncertainty of the effective point of measurement, the dose gradient across the chamber, the uncertainty of back‐scatter characteristics of construction materials, the dissimilarities of plastics and water, the number and magnitude of correction factors, among others. Many of these arguments and resulting disparities are answered by the implementation of the AAPM Task Group 51 (TG‐51) protocol for clinical reference dosimetry of high energy photon and electron beams.^14^

In addition, the AAPM TG‐21 calibration protocol allows both dosimetry systems to be used for both photon and electron beam calibration, but it recommends that low‐energy electron beams be calibrated using a parallel plate chamber. The TG‐51 protocol states that the annual calibration is to be performed only in water. Parallel‐plate chambers are again recommended for low‐energy electron calibrations, but they are specifically *excluded* from calibration of photon beams.

The arguments and recommendations issued by TG‐51 are based on valid physical principles, measured and/or calculated data, estimations of uncertainties, and personal or professional biases. Expressing the personal or professional preferences of the authors, it is suggested that the major advantages of the two dosimetry systems are: (i) For the Holt/polystyrene system: The Holt chamber in polystyrene is as close to a pure Bragg‐Gray system as we have available, in that the chamber and phantom form a homogeneous polystyrene medium enclosing a thin gas cavity with a virtually negligible wall. Foreign materials including electrical leads are minimal, and the cavity is well guarded laterally. (ii) For the Farmer/water system: Water is universally accepted as the standard phantom material, and Farmer‐type chambers have proved to be extremely reliable in this medium. The Farmer‐type chambers with their airlike (graphite) or waterlike (acrylic) walls are also reasonable approximations of a Bragg Gray cavity, and they have been studied extensively, both empirically and theoretically, for some 30 years. The TG‐51 protocol, however, attempts to minimize the differences that may result from the use of the Farmer‐type and Holt parallel plate chambers in water and polystyrene and the TG‐21 protocol. Even after more than two years since the publication of TG‐51, the vast majority (~70%) of the clinics monitored by the RPC were still using the TG‐21 protocol.

This work presents a direct comparison of the absolute dose measurements for the two dosimetry systems for numerous photon and electron energies (${}^{60}\text{Co}$ *γ* rays to 18 MV × rays and from 6–20 MeV electrons) using the TG‐21 protocol. An attempt to explain the observed differences is made and an argument is made for the implementation of TG‐51 as a solution to resolving the observed differences in electron beam dosimetry.

## II. MATERIALS AND METHODS

### A. Equipment

The RPC's ionization measurements were made in a water phantom using two commercially available Farmer‐type cylindrical 0.6‐cm^3^ ion chambers, an NEL model 2571 (Nuclear Enterprises, Ltd., Fairfield, NJ), and a PTW model N23333 (PTW/Nuclear Associates, Carle Place, NY), connected to a Keithley model 602 electrometer (Keithley Instruments, Cleveland, OH), modified to incorporate a digital display. The MSK ionization measurements were made with a Holt Memorial 1.0 cm^3^ parallel‐plate ion chamber in a polystyrene phantom connected to a Memorial two‐channel electrometer, model RDL‐3 (Memorial Sloan‐Kettering Cancer Center, New York, NY). The smallest dimension of both phantoms was at least 25 cm.

Machine output at $d_{\max}$ depth in a 10 cm×10 cm field size at 100 cm source to surface distance (SSD), was determined using both systems for a series of photon and electron beams from linear accelerators and Theratron 80 and Theratron 780 ${}^{60}\text{Co}$ units (AECL/Theratronics International Ltd., Kanata, Ontario, Canada). The accelerator models included Varian Clinac 6/100, Clinac 600C, Clinac 18, Clinac 20, and Clinac 2100C (Varian Associates, Palo Alto, CA), Therac 20 (AECL/Theratronics International Ltd.), and Siemens Mevatron XII (Siemens Medical Corp., Iselin, NJ). The photon and electron energies ranged from ${}^{60}\text{Co}$ to 15 MV and from 6 to 20 MeV respectively.

### B. Dosimetry system quality assurance

The exposure calibration factor $\left(N_{x})\right)$ and the electrometer factor for the RPC's dosimetry systems were verified immediately before each trip to MSK. This verification was performed by intercomparison with the RPC's working standard, a modified NEL 2505/3A cylindrical chamber, in a ${}^{60}\text{Co}$ beam. The RPC's chambers have factors that are traceable to the national standards, yet are independent of the standards of any specific Accredited Dosimetry Calibration Laboratory (ADCL). The Memorial Holt parallel‐plate ion chambers used by MSK have $N_{x}$ factors assigned to them by the MSK ADCL. The MSK factor was assigned in a ${}^{60}\text{Co}$ beam with the parallel plate chamber placed in‐air. The MSK parallel‐plate ion chamber factors were then verified every six months in a ${}^{60}\text{Co}$ beam and a 6 MV beam against MSK's standard dosimetry system. An intercomparison of the RPC's cylindrical chambers and the Holt parallel‐plate chamber was performed in air in a ${}^{60}\text{Co}$ beam and at depth in a polystyrene phantom in a 6 MV beam at MSK.

### C. Absorbed‐dose calculations

The RPC and MSK both used the TG‐21 protocol to determine the absorbed‐dose rate at the reference point from high‐energy photon and electron beams. The RPC applied two modifications to the protocol which have been fully described previously.^15^ ^,^ ^16^ Specifically, for photon beams, an additional correction for the effects of a plastic protective cap, $P_{\text{cap}}$, was applied to the calculations.^15^ For electron beams the RPC applied a 2‐mm shift upstream from the center of the cylindrical chamber to the effective point of measurement for all measurements, even though TG‐21 does not recommend this correction at $d_{\max}$.^16^ The MSK calculations using the Holt parallel‐plate ion chamber followed the TG‐21 and TG‐39 protocols^17^ explicitly.

## III. RESULTS AND DISCUSSION

The results of the output comparisons discussed above are summarized in Tables I and II, as ratios of the absorbed doses determined by the RPC versus the absorbed doses determined by MSK. As such, they represent the absorbed doses determined by the cylindrical system divided by those determined by the parallel‐plate system. The absorbed dose values presented in Tables I and II are full TG‐21 calculations to $d_{\max}$ depth for a reference 10 cm×10 cm field size. Previous work comparing Farmer‐type chambers to the Holt parallel plate chamber typically compared only the $N_{\text{gas}}$ values determined in the same medium (usually polystyrene) and not the absolute absorbed dose.^6^ ^,^ ^10^ ^,^ ^18^ ^,^ ^19^ During the 1985 on‐site dosimetry visit to MSK, the RPC reviewers used two NEL 2505/3A chambers (Serial Nos. W3‐3 and W3‐5) for all machine output measurements. Although it was not noted precisely which Holt parallel‐plate chambers MSK used for output checks, the chamber intercomparison measurements made in the ${}^{60}\text{Co}$ beam between the two RPC chambers and the MSK 155 YP chamber were in excellent agreement (within 0.2%). During the 1993 visit, the RPC reviewers used two chambers, an NEL 2571 and a PTW N23333 for measurements, and MSK used four Holt parallel‐plate chambers. The chamber intercomparisons yielded an $N_{x}$ ratio (RPC/MSK) of 0.999 in a ${}^{60}\text{Co}$ beam and 1.007 in a 6 MV x‐ray beam.

**Table I:** RPC/MSK ratios for photon output measurements for two RPC dosimetry review visits comparing cylindrical and parallel plate ionization chambers. Abbreviations: RPC, Radiological Physics Center; MSK, Memorial Sloan‐Kettering Cancer Center.

1985 visit Therapy unit	Energy	RPC chamber NEL # W3‐5	RPC chamber NEL # W3‐3	MSK chamber
Theratron 780	Co‐60	—	1.005
Theratron 80	Co‐60	—	1.009	(data not captured)
Clinac 20	15	1.010	—
Therac 20	10	1.013	—
Therac 20	18	1.000	—
Mevatron XII	10	1.010	—
mean	value (SD)	1.008 (±0.005)		

1993 visit Therapy unit	Energy	RPC chamber NEL # 1541	RPC chamber PTW # A243	MSK chamber
Theratron 780	Co‐60	0.998	—	MPK 163
Clinac 6/100 (Rm 242)	6	—	0.992	MPK 163
Clinac 600C (Rm 441)	6	—	1.007	MPK 163
Clinac 600C (Rm 442)	6	—	1.004	MPK 163
Clinac 600C (Rm 443)	6	—	0.999	MPK 160
Clinac 2100C (Rm 445)	6	1.002	—	MPK 134
“	15	1.002	—	MPK 134
Clinac 2100C (Rm 245)	6	—	0.995	MPK 154
“	15	—	1.001	MPK 154
Clinac 18	10	—	0.999	MPK 163
Clinac 20	15	1.010	—	MPK 163
mean	Value (SD)	1.001(±0.005)		

**Table II:** RPC/MSK ratios for electron output measurements for two RPC dosimetry review visits comparing cylindrical and parallel plate ionization chambers.

1985 visit Therapy unit	Energy	RPC chamber NEL # W3‐5	RPC chamber NEL # W3‐3	MSK chamber
Clinac20	6	0.975		
“	9	0.985		
“	12	0.975		
“	16	0.986	(data not collected)	(data not collected)
Therac 20	6	0.987		
“	9	0.991		
“	13	0.985		
“	17	1.011		
“	20	0.987		
Mevatron XII	6	0.986		
“	11	1.006		
mean	value (SD)	0.989 (±0.011)		

1993 visit Therapy unit	Energy	RPC chamber NEL #1541	RPC chamber PTW #A243	MSK chamber
Clinac 2100C (Rm 445)	6	0.996	—	MPK 160
“	9	0.994	—	”
“	12	0.991	—	”
“	16	0.982	—	”
“	20	0.997	—	”
Clinac 2100C (Rm 245)	6	—	0.969	MPK 154
“	9	—	0.980	”
“	12	—	0.981	”
“	16	—	0.982	”
“	20	—	0.989	”
Clinac 18	6	—	0.999	MPK 163
“	9	—	0.988	”
“	12	—	0.983	”
“	15	—	0.982	”
“	18	—	0.988	”
Clinac 20	9	0.980		”
“	12	0.981		”
“	16	0.974		”
“	20	0.982		”
mean	value (SD)	0.985 (±0.009)		

**Figure 1:**
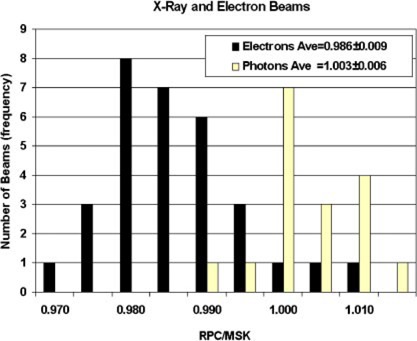
Frequency histogram of the Radiological Physics Center/Memorial Sloan‐Kettering dose ratios for both photons (light bars) and electrons (dark bars) for the two RPC on‐site dosimetry review visits.

Table I lists the ratios (RPC/MSK) for photon beam output in the six photon beams measured in 1985, which included four different beam qualities and six different units. The average ratio was 1.008±0.005. In 1993 the average ratio (RPC/MSK) for photon beam output for 11 photon beams, including four different beam qualities from nine different units, was 1.001±0.005 (Table I). The data in Fig. 1 (light bars) represent a frequency histogram of the dose ratio (RPC/MSK) for photon beams from both the 1985 and 1993 visits. Between the 1985 and 1993 visits, the RPC changed its exposure standard by 0.9%, which is consistent with the difference between the mean RPC/MSK ratios of photon output measured during the two visits.

The data in Table I indicate very little difference between the Farmer‐type and Holt parallel plate chamber dosimetry systems in the two different media for megavoltage photon beams. Therefore, conversion to the TG‐51 protocol should result in the same magnitude of change for the parallel plate system as would be expected from previously published values^20^ ^–^ ^25^ for the Farmer‐type chamber system (NEL 2571 and PTW N23333 chambers), i.e., approximately 0.5−1.2% depending on the energy. However, the TG‐51 protocol explicitly prohibits the use of parallel plate chambers and plastic media for the calibration of photon beams.

Table II lists electron beam output comparisons that were made for 12 electron beams in 1985 and 19 electron beams in 1993, ranging in energy from 6 to 20 MeV from six therapy units. The average electron RPC/MSK ratios were 0.989±0.011 and 0.985±0.009 for the 1985 and 1993 visits, respectively. The frequency histogram of the dose ratio, RPC/MSK for electrons (dark bars) and the average percent difference for the two visits are also shown in Fig. 1. These data are in direct contrast to the data published by Krithivas and Rao^10^ who showed no difference between the Farmer‐type and Holt parallel plate chambers. Two differences between the Krithivas data and the data presented here are (1) Krithivas determined the doses in the same media for both chamber systems, not water versus polystyrene and (2) the Krithivas cylindrical chamber dose measurements were determined without incorporating the correct shift to the effective point of measurement which will influence the dose measurements at the low electron energies.

The measured data presented here suggest that the differences observed in the measurements between the various Farmer chambers and the Holt parallel‐plate chamber are not a function of energy but of radiation type, i.e., electron versus photon beam. The average ratio (RPC/MSK) of beam outputs was 0.987±0.011 for electrons and 1.003±0.006 for all photon beams, which suggested that the Holt parallel‐plate chamber measures an output that is on average approximately 1.3% higher for electron beams than the photon beam output measured by Farmer‐type chambers. The individual output ratios used to calculate the average ratios quoted above for the photon and electron beam outputs vary. The ranges of the electron and photon values were 0.969−0.999 excluding what appears to be two outliers (1.006 and 1.011) and 0.992−1.013, respectively. The variation in the electron beam output ratios seems to be greatest at the lowest energy (6 MeV) where the precision of the measurement is dependent on chamber positioning. The variation in beam output ratios for the remaining electron energies is approximately ± 1.5% for each energy as might be expected for the combined uncertainties of two calibrations. Thus, there is on average a 1.7% difference between the two systems for electrons and photons with the electron beam output ratios tending to be smaller than the photon output ratios. This observed difference between electrons and photons can be related to the results of Kubo *et al.*,^6^ ^,^ ^18^ who reported a difference of 1.7 to 1.9 % in the $N_{\text{gas}}$ value when the value was derived from readings with Farmer‐type chambers in‐air in ${}^{60}\text{Co}$ beams and in polystyrene in high‐energy electron beams. Our results agree with this, but they were derived from measurements of a much wider range of photon and electron beam energies and our data include differences in phantom material.

Others have made efforts to seek more appropriate values for $P_{\text{repl}}$ and $P_{\text{wall}}$ ^19^ for cylindrical chambers that in turn would render a different $N_{\text{gas}}$ value for a parallel‐plate chamber derived from that of a cylindrical chamber.^17^ In addition to the uncertainty in the $P_{\text{repl}}$ and $P_{\text{wall}}$ values, another source of uncertainty that may assist in explaining the electron dose differences is the electron beam water to polystyrene fluence correction, $\phi_{\text{poly}}^{w}$. Ding *et al.* ^25^ reported that there are large variations in the fluence correction factors published in the literature for polystyrene. There even exist differences in the fluence correction factors between the TG‐21 and TG‐25 protocols.^26^ The uncertainty in this fluence correction may play a significant role in explaining the differences noted in the measurement of the absorbed dose in water versus polystyrene. The data discussed above indicate that the dose measured in polystyrene with the Holt parallel plate chamber has a larger uncertainty than that measured in water with a cylindrical chamber. Which of these parameters is responsible for the measured discrepancy and to what degree remains to be clarified, but they probably all contribute to the observed differences.

A simple method for eliminating the observed dose differences for the electron beams is the implementation of the TG‐51 protocol, as it does not allow the use of polystyrene as a calibration medium. The required use of water as the calibration medium in TG‐51 eliminates the uncertainty in the polystyrene to water fluence correction factor. Clinics that have in the past used a Holt parallel plate chamber system in a polystyrene phantom and switch to a cylindrical chamber system to calibrate their electron beams will see very little difference in the electron beam dose rates between their TG‐21 calibrations and the new TG‐51 calibrations. Based on the differences noted in this work for the Holt parallel plate and a cylindrical chamber and the expected TG‐51 to TG‐21 ratios for electron beams, the two differences should nearly compensate so that the new TG‐51 dose rate is within 1% of the TG‐21 dose rate as determined by the cylindrical chamber. The energy dependence of the TG‐51 to TG‐21 ratios for electron beams will also affect the resulting TG‐51 electron calibration since the ratio increases with energy and our parallel plate to cylindrical chamber ratios did not depend on energy. Physicists can use the data presented here to help explain why the percent change in electron beam output when converting from TG‐21 to TG‐51, switching from using the MPPK to a cylindrical chamber, respectively, does not agree with published values.^20^ ^–^ ^24^

## IV. CONCLUSIONS

The data presented here are the most thorough comparison published to date of the two dosimetry systems used most frequently in the USA. The Holt parallel‐plate chamber in polystyrene and Farmer‐type chambers in water agree on average to within 1% on the determination of photon‐absorbed dose in water for energies between ${}^{60}\text{Co}$ and 18 MV. For electron beam dose determinations, however, the Holt parallel‐plate chamber differs from the Farmer‐type chamber by an average of 1.3%. Even though the reasons for the observed differences in electron beam calibrated dose rates are not explicitly known, they can be essentially eliminated, i.e., to within +/−1% of the values determined with cylindrical chambers, by implementation of the TG‐51 calibration protocol.

## ACKNOWLEDGMENTS

This work was supported by Public Health Service Grant No. CA10953 awarded by the National Cancer Institute, United States Department of Health and Human Services. We would like to thank Elizabeth Siller for typing the many drafts of this manuscript.
